# Epigenetic clock indicates accelerated aging in glial cells of progressive multiple sclerosis patients

**DOI:** 10.3389/fnagi.2022.926468

**Published:** 2022-08-24

**Authors:** Lara Kular, Dennis Klose, Amaya Urdánoz-Casado, Ewoud Ewing, Nuria Planell, David Gomez-Cabrero, Maria Needhamsen, Maja Jagodic

**Affiliations:** ^1^Department of Clinical Neuroscience, Center for Molecular Medicine, Karolinska University Hospital, Karolinska Institutet, Stockholm, Sweden; ^2^Neuroepigenetics Laboratory, Navarrabiomed, Hospital Universitario de Navarra (HUN), Universidad Pública de Navarra (UPNA), IdiSNA, Pamplona, Spain; ^3^Translational Bioinformatics Unit, Navarrabiomed, Hospital Universitario de Navarra (HUN), Universidad Pública de Navarra (UPNA), IdiSNA, Pamplona, Spain; ^4^Unit of Computational Medicine, Department of Medicine, Center for Molecular Medicine, Karolinska University Hospital, Karolinska Institutet, Stockholm, Sweden; ^5^Mucosal and Salivary Biology Division, King’s College London Dental Institute, London, United Kingdom; ^6^Biological and Environmental Sciences and Engineering Division, King Abdullah University of Science and Technology, Thuwal, Saudi Arabia

**Keywords:** multiple sclerosis, DNA methylation, aging, epigenetic clock, brain, glial cells, neurons

## Abstract

**Background:**

Multiple sclerosis (MS) is a chronic inflammatory neurodegenerative disease of the central nervous system (CNS) characterized by irreversible disability at later progressive stages. A growing body of evidence suggests that disease progression depends on age and inflammation within the CNS. We aimed to investigate epigenetic aging in bulk brain tissue and sorted nuclei from MS patients using DNA methylation-based epigenetic clocks.

**Methods:**

We applied Horvath’s multi-tissue and Shireby’s brain-specific Cortical clock on bulk brain tissue (*n* = 46), sorted neuronal (*n* = 54), and glial nuclei (*n* = 66) from post-mortem brain tissue of progressive MS patients and controls.

**Results:**

We found a significant increase in age acceleration residuals, corresponding to 3.6 years, in glial cells of MS patients compared to controls (*P* = 0.0024) using the Cortical clock, which held after adjustment for covariates (*P_*adj*_* = 0.0263). The 4.8-year age acceleration found in MS neurons (*P* = 0.0054) did not withstand adjustment for covariates and no significant difference in age acceleration residuals was observed in bulk brain tissue between MS patients and controls.

**Conclusion:**

While the findings warrant replication in larger cohorts, our study suggests that glial cells of progressive MS patients exhibit accelerated biological aging.

## Introduction

Multiple sclerosis (MS), a leading cause of non-traumatic neurological disability among young adults, is a progressive inflammatory disease of the central nervous system (CNS) characterized by focal demyelination and subsequent neuro-axonal degeneration gradually impinging the CNS (Filippi et al., 2018). The vast majority (85%) of MS patients present with an early inflammatory relapsing-remitting form of the disease with periodic autoimmune attacks of the myelin and ensuing neuro-axonal damage. Most patients will eventually convert to a progressive form of MS, namely secondary progressive MS (SPMS), presenting with limited remyelination and an unabating increase in neurodegeneration and disability without remission. A small fraction of patients, approximately 10%, encounters a progressive course of disease already from the onset, referred to as primary progressive MS (PPMS). While major progress has been made in understanding and treating the peripheral immunopathogenesis occurring at the early stages of MS, CNS-restricted mechanisms underlying later progressive stages remain puzzling (Ciotti and Cross, 2018). This gap of knowledge considerably hampers treatment options for progressive MS patients, and further demands concerted efforts to address CNS-confined processes in MS.

Accumulating evidence implies that the accrual of irreversible disability may be age dependent. The ability to recover from relapse decreases with age (Cossburn et al., 2012; Kalincik et al., 2014) and the risk to enter progressive MS stage increases with age, irrespective of the initial disease course (Confavreux and Vukusic, 2006; Tutuncu et al., 2013). Accordingly, aging-related processes have been identified in MS patients. In the periphery, shortening of telomere length in leukocytes has been associated with greater disability, lower brain volume, higher relapse rate, and more rapid conversion to progressive MS (Buhring et al., 2021). Premature cellular senescence impairing remyelination potential has been identified in the brain of progressive MS patients as well (Nicaise et al., 2017, 2019). Moreover, brain age estimation using cross-sectional and longitudinal neuroimaging has uncovered accelerated aging of the brain in patients, with brain atrophy and white matter lesion load being the strongest predictors of the progressing aging measure (Hogestol et al., 2019). Overall, accelerated aging in the CNS of MS patients, likely arising from chronic exposure to inflammation, has been proposed as a putative mechanism underpinning the relentless decline in the CNS ability to repair and restore its functional capacity observed at progressive stages of disease (Kular and Jagodic, 2020).

DNA methylation (DNAm)-based epigenetic clocks stand among the most reliable estimators of the biological age, and as such are regarded as promising biomarkers of aging and organ function. The most established epigenetic clocks, that is, Horvath (Horvath, 2013), Hannum (Hannum et al., 2013), and PhenoAge (Levine et al., 2018), strongly correlate with aging-related outcomes, such as all-cause mortality, physical functioning, and aging-related pathologies including cognitive decline (Marioni et al., 2015; Levine et al., 2018). Among them, the multi-tissue predictor of DNAm age by Horvath has been used in bulk brain tissue of donors affected by neurological disorders, such as Huntington’s and Alzheimer’s diseases, and has shown an association with neuropathological markers of neurodegeneration (Levine et al., 2015; Horvath et al., 2016). Yet, studies have reported spurious associations between DNAm age and clinical readouts in certain samples, thus revealing a potential bias of these blood-trained clocks which might interfere with the analysis of CNS tissue of older donors (Zhang et al., 2019). Moreover, blood-brain paired analyses have reported a modest correlation between the respective DNAm ages in blood compared to brain tissue (Grodstein et al., 2020), reinforcing the need to investigate aging-related mechanisms of neurological diseases *in situ*, in the primarily affected tissue. The Cortical clock, trained exclusively on post-mortem human cortical samples, has been developed to further improve the estimation of DNAm age in brain tissue (Shireby et al., 2020). Both Horvath’s and the Cortical clock have been trained on samples from non-diseased individuals, using elastic net regression for the identification of relevant CpGs among the vast amount of analyzed CpG probes from the Illumina 450K/EPIC arrays. However, the Cortical clock has been shown to yield a higher predictive value and outperformed Horvath’s clock in cortical bulk brain specimens (Grodstein et al., 2020; Shireby et al., 2020).

We have previously reported differences in DNAm age measures in peripheral blood and sorted cells of MS patients and healthy controls (Theodoropoulou et al., 2019). Here, we aim to utilize genome-wide DNA methylation profiles in CNS cells from case-control cohorts (Huynh et al., 2014; Kozlenkov et al., 2014; Gasparoni et al., 2018; Kular et al., 2019, 2022) to examine age acceleration in bulk brain tissue, neurons, and glial cells of MS patients in comparison to non-neurological controls.

## Materials and methods

### Study samples

Our study included a total of five DNA methylation datasets generated from bisulfite-treated DNA derived from post-mortem bulk brain and sorted nuclei fractions of MS patients and non-neurological controls (NNC) using Illumina Infinium HumanMethylation450 (450K) or EPIC platforms. Details are listed in Table 1. In brief, bulk brain 450K DNA methylation data from MS patients and controls were retrieved from a study by Huynh et al. (2014) (GEO: GSE40360) and comprise 46 normal-appearing white matter (NAWM) and control white matter brain samples, as previously described (Huynh et al., 2014). DNA methylation data from 32 neuronal (NeuN^+^) (GEO: GSE119532) and 44 glial (NeuN^–^) nuclei (GEO: GSE166207) samples of MS patients and controls were generated using 450K and EPIC, respectively, as previously described (Kular et al., 2019, 2022). The disease course of all cases was classified as progressive MS at the time of death. All neuronal and glial datasets originate from patients with secondary progressive SPMS and the majority (63%) of bulk NAWM samples were obtained from SPMS individuals with additional samples coming from patients with PPMS and chronic progressive MS (Table 1). To cover a wider age range and to counteract collinearity of age and clinical group (MS/NNC) in the neuronal and glial cohort (*P*_*Neurons*_ = 1.31 × 10^–5^, *P*_*Glia*_ = 1.44 × 10^–6^ with two-sided two-sample *t*-test), we included additional 450K DNA methylation data generated from neuronal and glial nuclei of NNC donors, from Kozlenkov et al. (2014) (GEO: GSE50798, *n* = 6 individuals) and Gasparoni et al. (2018) (GEO: GSE66351, *n* = 16 individuals). These external datasets provide glial and neuronal data from the same donors. Across all included studies, neuronal and glial nuclei were sorted by fluorescence-activated cell sorting using neuronal nuclear antigen (NeuN) as a neuronal marker. To provide an example of Cortical clock performance on non-nervous tissue, we included whole blood 450K methylation data, previously described in Kular et al. (2018) and Theodoropoulou et al. (2019) (GEO: GSE106648).

**TABLE 1:** Cohort characteristics.

Platform GEO accession	Glia^#^ *Illumina EPIC/450K GSE166207, GSE50798, GSE66351*		Neurons^#^ *Illumina 450K GSE119532, GSE50798, GSE66351*		Bulk brain *Illumina 450K GSE40360*	

Group	*NNC*	*MS*	*NNC*	*MS*	*NNC*	*MS*
N donors	36	13	34	14	19	27
MS disease course (N)	−	SPMS (13)	−	SPMS (14)	−	SPMS (17), PPMS (7), CP (3)
Sex ratio (F/M)	11/25	11/2	10/24	10/4	7/12	17/10
Age (m/SD)	64.8/24.5	56.7/13.5	63.8/25.1	55.2/13.3	66.3/8.5	55.3/9.9
PMI (m/SD)*	26.2/21.7	17.5/7.9	25.5/21.9	16.6/8.2	16.7/5.6	16.2/6.7
N brain samples	45	21	37	18	19	27
N sample types	−	AL (3)	−	AL (2)	−	NAWM (27)
	−	CL (2)	−	CL (5)	−	−
	−	NAWM (16)	−	NAWM (11)	−	−

N, number; F, female; M, male; PMI, post-mortem interval; m, sample mean; SD, standard deviation; NNC, non-neurological controls; MS, multiple sclerosis; SPMS, secondary progressive MS; PPMS, primary progressive MS; CP, chronic progressive; NAWM, normal-appearing white matter; AL, active lesion; CL, chronic lesion. ^#^The glia cohort (Kozlenkov et al., 2014; Gasparoni et al., 2018; Kular et al., 2022) and the neuronal cohort combine three independent datasets (Kozlenkov et al., 2014; Gasparoni et al., 2018; Kular et al., 2019). *PMI info is missing for 15 donors including 3 bulk brain, 10 neuronal, and 14 glial samples.

### Preprocessing of DNA methylation data

All analyses were conducted in the R statistical environment (version 4.0.5^1^) with the RStudio software (version 1.4.1106^2^), if not stated otherwise. For all datasets, raw data were retrieved, that is, the intensity of the fluorescence signal for 5-methylated and unmethylated cytosines. The *readGEORawFile* function from the *minfi* package (version 1.40.0) was used to import the raw data from studies by Huynh et al. (2014) and Kozlenkov et al. (2014). The *pfilter* function from the *wateRmelon* package (version 2.0.0) was used to exclude CpG probes with >5% of samples with detection *P* > 0.05. Data from the bulk brain cohort, the combined glial cohort, the combined neuronal cohort, and the blood cohort were normalized separately using the *dasen* function from the *wateRmelon* package to perform background and Type I/Type II bead-type correction. The *combineArrays* function from the *minfi* package was used to merge EPIC- and 450K-derived data for GSE166207, GSE50798, and GSE66351. Neuronal and non-neuronal proportions in bulk brain tissue were estimated with the *meffil.estimate.cell.counts.from.betas* function from the *meffil* package (version 1.1.1), which deploys the Houseman deconvolution algorithm using the dorsolateral prefrontal cortex as reference DNA methylation data (Houseman et al., 2012; Guintivano et al., 2013).

### DNA methylation age estimation and linear models

For DNAm age estimation, we applied the *agep* function from the *wateRmelon* package. In total, 353 and 348 CpG probes were considered for the Horvath and Cortical clock, respectively, of which five probes are shared. The EPIC platform lacks 19 Horvath’s clock CpG probes used for DNAm age estimation, thus these probes could not be included in our analysis for glial cells. For this reason and due to P-value filtering, a slightly reduced set of CpG probes was included in the final *agep* calculation of DNAm age and we ensured that the age estimation was not compromised by the 19 missing probes, as shown in bulk brain and neuronal material (Supplementary Figure 1).

For the calculation of age acceleration residuals (AAR), the *resid* and *lm* functions from base R were used, with DNAm age as the dependent variable and chronological age as the independent variable in the linear model. For AAR calculation in bulk brain tissue, predicted neuronal proportions were included as a second independent variable to account for the reported association of decreased neuronal proportions and higher DNAm age. By definition, the AAR is independent of the donors’ chronological age (and neuronal proportions). Linearity, normality, and homoscedasticity of residuals derived from the linear model were validated graphically with the *plot* and *lm* functions from base R.

### Statistical analysis

Normality and equal variances of AAR, when stratified for clinical groups (MS/NNC), were tested with Shapiro–Wilk and Bartlett’s test. The mean AAR of MS and NNC clinical groups was compared with a two-sided two-sample *t*-test. If equal variances could not be assumed, a two-sided Welch’s *t*-test was performed instead.

Covariate adjusted P-values for the comparison of AAR between clinical groups were calculated with a multivariate analysis of the covariance linear model (MANCOVA). The covariates included in each analysis were: sex and post-mortem interval (PMI) for the bulk brain cohort; sex, PMI, and sample replicate status (i.e., different brain samples taken from the same individual) for the neuronal cohort; and sex, PMI, sample replicate status, and principal component 1 (PC1) as a proxy for glial cohort origin, as the use of glial cohort origin as a categorical variable would interfere with the clinical group variable since external datasets only include control samples.

Brain region-specific AAR differences were analyzed with a Tukey HSD test without adjustments, as sex and PMI had no significant influence on AAR (*P*_*Sex*_ = 0.47, *P*_*PMI*_ = 0.48) in these subsets of samples from the same donors. If data from the same individuals were compared, a two-sided paired *t*-test was performed. In the study of age acceleration in subgroups stratified according to sex, we performed a Tukey HSD test on the residuals of the MANCOVA model (covariates for glial cohort: PMI, replicate status, PC1; covariates for neuronal cohort: PMI, replicate status). The significance level for all tests was set to *P* = 0.05.

## Results

### Performance of Cortical and Horvath’s clock DNA methylation age estimations for brain-derived tissue

**FIGURE 1:**
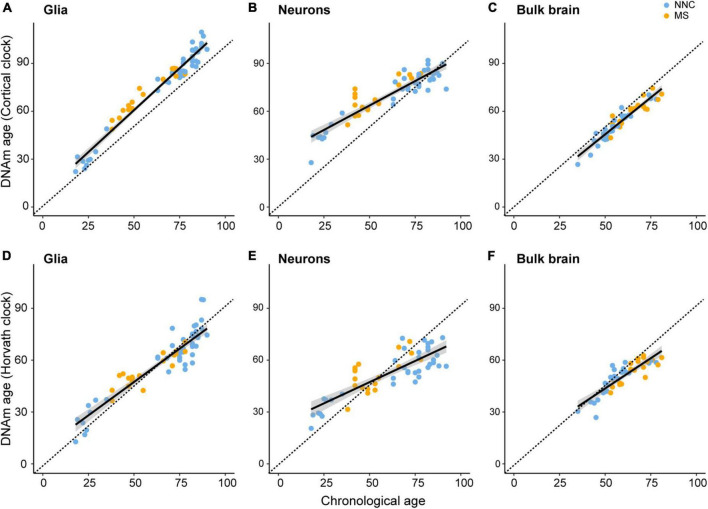
Linear models of Cortical and Horvath’s clocks applied to glial cells, neurons, and bulk brain tissue. Linear models of regressing estimated DNAm age on chronological age with the DNAm age estimated with the Cortical clock [upper panels **(A–C)**] or Horvath’s clock [lower panels **(D–F)**]. Regression of DNAm age on chronological age was performed in glial nuclei **(A,D)**, neuronal nuclei **(B,E),** and bulk brain tissue **(C,F)** of multiple sclerosis (MS) patients and non-neurological controls (NNC). The dashed line represents the x = y bisector and the black line represents the linear regression line of best fit. For information on model characteristics, see Table 2. Data were retrieved from Huynh et al. (2014), Kozlenkov et al. (2014), Gasparoni et al. (2018), and Kular et al. (2019, 2022).

To assess biological aging in brain cells of MS patients and controls, we first sought to compare epigenetic clocks that have been previously used in brain samples, that is, the multi-tissue Horvath’s clock and the brain-restricted Cortical clock, in our study samples composed of bulk NAWM (*n* = 46), sorted neuronal (*n* = 54), and glial nuclei (*n* = 66) from post-mortem brain material (Table 1). In the bulk brain and neuronal nuclei, the Cortical clock model showed higher R^2^ values and lower mean absolute error (MAE) indicative of a more accurate estimation of epigenetic age over the whole lifespan (Figures 1A–C and Table 2), confirming previous reports (Shireby et al., 2020). While a higher R^2^ value could also be observed using the Cortical clock compared to Horvath’s clock in the glial nuclei, higher MAE denoted the systematic overestimation of DNAm age in glial cells with the Cortical clock (*MAE*_*Horvath*_ = 6.88 vs. *MAE_*Cortical*_* = 11.48, Figure 1D and Table 2). This overestimation is typically addressed by using the aging residuals instead of the absolute difference between predicted and chronological age, as done in the subsequent analyses of this study. Unsurprisingly, the estimation of DNAm age from blood samples was more accurate with the multi-tissue Horvath’s clock as opposed to the Cortical clock (Table 2). Moreover, we observed a higher intercept in the neuronal cohort as opposed to the bulk brain and glial cohorts with both clocks (Table 2), which implies an overestimation of younger donors’ DNAm age in neurons specifically by both clocks. A general underestimation of the DNAm age of older donors in the bulk brain and neuronal cohorts could be observed with Horvath’s clock (Figures 1E,F). Such effect could not be detected when Horvath’s clock was applied to blood samples (Supplementary Figure 2). Of note, Metascape Gene Ontology Analysis (Zhou et al., 2019) of CpG-annotated genes by both clocks yielded common but also unique pathways enriched in either one clock or the other (Supplementary Table 2). This implies that different aspects of aging might be captured by each clock, which can be reflected by differences in AAR values generated by one clock compared to the other (Supplementary Figure 3).

**TABLE 2:** Characteristics of linear regression of DNAm age estimations from Horvath’s and Cortical clock on chronological age.

	Horvath’s clock					Cortical clock				

	*R* ^2^	*P*	*MAE*	*Intercept*	*Slope*	*R* ^2^	*P*	*MAE*	*Intercept*	*Slope*
Glia	0.86	< 2.2 × 10^–16^	6.88	8.75	0.77	0.95	< 2.2 × 10^–16^	11.48	8.14	1.05
Neurons	0.67	1.79 × 10^–14^	11.9	22.72	0.49	0.82	< 2.2 × 10^–16^	9.76	32.98	0.61
Bulk	0.74	1.68 × 10^–14^	9.33	8.63	0.69	0.88	< 2.2 × 10^–16^	5.23	-0.52	0.92
Blood	0.87	< 2.2 × 10^–16^	6.53	16.18	0.77	0.78	< 2.2 × 10^–16^	8.11	25.75	0.57

R^2^, coefficient of determination; P, P-value for the F-statistic; MAE, mean absolute error.

Taken together, these results indicate that aging residuals of brain tissue-derived samples are more accurately estimated with the Cortical clock compared to Horvath’s clock.

### Exploration of confounding factors for DNA methylation age and age acceleration residuals estimates

DNA methylation estimates can be biased by both technical and biological confounders, the latter commonly referred to as covariates. To address potential technical confounders, we first explored putative batch effects and found minimal variation in DNAm age estimation between replicates loaded on different platforms or slides (Supplementary Figures 4A,B), confirming DNAm age reproducibility. We examined different data normalization methods as well, that is, dasen- (originally used for training the Cortical clock), BMIQ-, and Noob-based normalization, and found that dasen-normalized beta values yielded the highest R^2^ value in the linear model of both clocks compared to no normalization or other normalization methods, prompting us to opt for dasen normalization (Supplementary Figure 5). While the use of three independent datasets in each cell type-specific cohort greatly improved the collinearity between age and clinical group (*P*_*Neurons*_ = 0.041 and *P*_*Glia*_ = 0.055 with a two-sided two-sample *t*-test), variation between datasets was visible only for the glial cohort, as shown by the PCA separation (*P*_*Dataset*_ = 2.4 × 10^–10^ with Kruskal–Wallis test) (Supplementary Figures 6A,B). This effect was taken into account in the MANCOVA model by including PC1 as a proxy for glial cohort origin and we ensured that PC1 for the glial cohort was not majorly confounded by other sample features (*P*_*Sex*_ = 0.109, *P*_*Anterior/Posterior*_ = 0.402, *P*_*NNC/MS*_ = 0.033 with Kruskal–Wallis test; *P*_*PMI*_ = 0.2, *P*_*Age*_ = 0.014 with Spearman’s correlation test). The inclusion of external control samples to cover a larger age range had a modest influence on the regression line used for AAR calculation (Supplementary Figures 6C,D).

**FIGURE 2:**
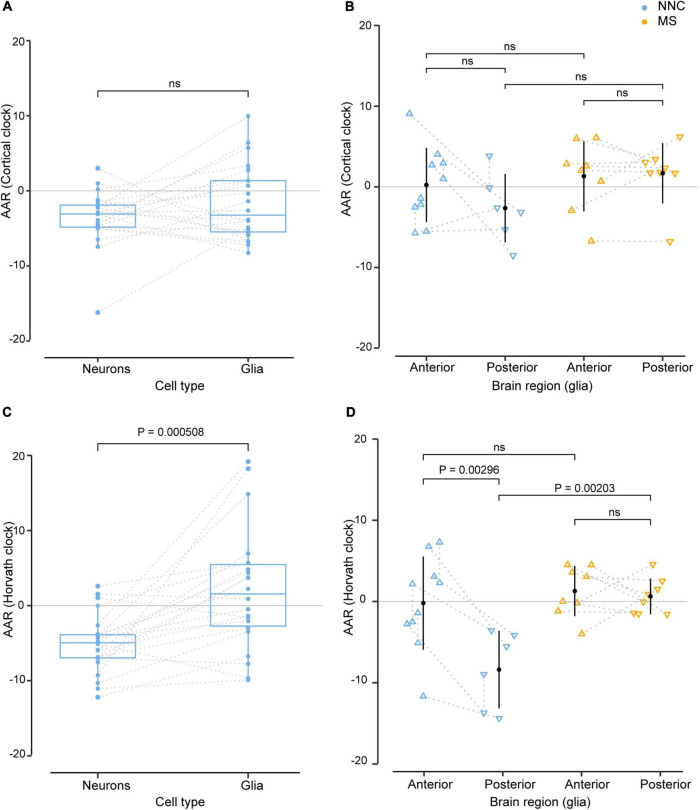
Effect of cell type and sample location from the same donors in the estimation of DNAm age. Age acceleration residuals (AAR) in glial cells and neurons sorted from the same control donors [left panels **(A,C)**] and in glial cells located in anterior and posterior regions of the brain of the same MS patients and controls [right panels **(B,D)**] estimated using the Cortical clock (upper panels) and Horvath’s clock (lower panels). Normality was tested with the Shapiro–Wilk test. **(A,C)** Sample differences were compared with a paired two-sample *t*-test. **(B,D)** Equal variances were tested with Bartlett’s test and sample means compared with a Tukey HSD test, correcting P-values for multiple testing with the single-step method **(B,D)**. The significance level for all tests was set to *P* = 0.05. Glia and neuron data from the same donors **(A,C)** were taken from Kozlenkov et al. (2014), Gasparoni et al. (2018), and anterior and posterior data in glia from the same donors [right panels **(B,D)**] were taken from Kular et al. (2022). The dotted line denotes the same donor. MS, multiple sclerosis; NNC, non-neurological control; ns, not significant.

We next explored putative covariates and addressed the effect of cell type (glial cells vs. neurons) and brain location (anterior vs. posterior) on AAR, using samples that were derived from the same donors. As shown in Figure 2, the DNAm clocks yielded different AAR depending on the cellular source. Results from both clocks indicated that, overall, glial cells displayed a higher variation of AAR compared to neurons (Figures 2A,C). While the Cortical clock yielded no significant difference between neurons and glial cells from the same donors (Figure 2A), the AAR of neurons was significantly lower than the AAR of glial cells using Horvath’s clock (Figure 2C). It should be noted that glial and neuronal AAR are derived from different models, suggesting that a portion of the observed differences might also arise from different AAR calculations (based on cell type-restricted regression lines). The effect of cellular heterogeneity can also be observed when comparing neuronal DNAm age estimations to the bulk brain tissue they originate from (Supplementary Figure 4C). Given the cell type-specific aging pace (Figure 2) and the fact that the DNAm age of brain samples with lower neuronal proportion is systematically overestimated (Shireby et al., 2020), we included algorithm-based estimations of neuronal proportion (Guintivano et al., 2013) as an independent variable in the bulk brain cohort’s linear model for the calculation of AAR. Varying AAR in samples from the same donor could also be observed according to the location of the brain regions along the anteroposterior axis (Figures 2B,D), which could be examined only in glial cells of Kular et al.’s dataset, due to insufficient data availability in the other glial, neuronal, and bulk datasets. This is particularly the case with Horvath’s clock applied to NNC individuals, where glial cells sorted from posterior brain regions showed significantly lower AAR compared to glial cells located in the anterior part of the brain (Figure 2D). Posterior glial cells of NNC also displayed significantly lower AAR compared to posterior glial cells of MS patients (Figure 2D).

Taken together, these findings suggest that glial cells and neurons of the same person age at a different pace and the location of brain samples might influence epigenetic aging measures.

### Glial cells of multiple sclerosis patients display DNA methylation age acceleration

**FIGURE 3:**
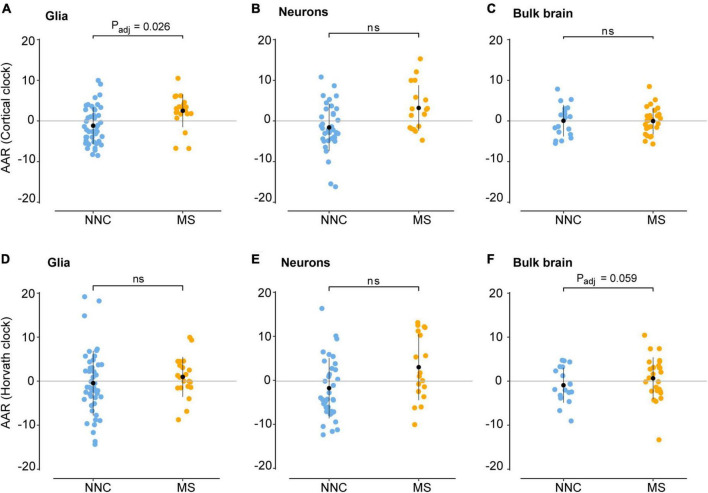
Age acceleration in glial cells, neurons, and bulk brain of MS patients and controls. Age acceleration residuals (AAR) in glial cells [left panels **(A,D)**], neurons [middle panels **(B,E)**], and bulk brain tissue [right panels **(C,F)**] of MS patients and controls were estimated using the Cortical clock (upper panels) and Horvath’s clock (lower panels). Normality was tested with the Shapiro–Wilk test. Equal variances were tested with Bartlett’s test. Sample means were compared with a two-sided two-sample *t*-test. In case of unequal variances **(D)**, Welch’s *t*-test was performed instead. Significance refers to covariate-adjusted P-values, with P_*adj*_ < 0.05 considered significant (for unadjusted P-values, see Table 3). Adjustment refers to sex, PMI, replicate status, PC1 (dataset of origin) in glia; sex, PMI, and replicate status in neurons; and sex, PMI, and neuronal cell proportions in the bulk brain. Data were retrieved from Huynh et al. (2014), Kozlenkov et al. (2014), Gasparoni et al. (2018), and Kular et al. (2019, 2022). MS, multiple sclerosis; NNC, non-neurological controls; ns, not significant.

We then examined the epigenetic clock-derived measures of DNAm age acceleration in bulk NAWM, glial, and neuronal samples of MS patients in comparison to NNC donors. The final models used for each cohort are described in the Materials and Methods section and the results are summarized in Table 3 and illustrated in Figure 3. In glial nuclei, AAR based on Cortical clock estimates revealed a significant difference between MS cases and controls after adjusting for covariates (*P*_*adj*_ = 0.0263, Figure 3A and Table 3). Of note, because Kozlenkov et al. did not report any PMI measures, these samples were not included in the adjustment. Nevertheless, a MANCOVA without PMI adjustment applied to all three glial datasets yielded similar significant results (*P*_*adj*_ = 0.0163). Overall, glial cells of MS patients were estimated to be on average 3.67 years older than those of NNC and 2.5 years older than what would be expected from the linear model (Table 3). The same tendency was observed with Horvath’s clock, but did not reach significance after adjusting for covariates (Δ*AAR_*MS–NNC*_* = 1.41, *P*_*adj*_ = 0.083, Table 3).

**TABLE 3:** Summary statistics of AAR comparisons between MS patients and controls.

	Horvath’s clock				Cortical clock			

	*m*_*NNC*_	*m*_*MS*_	*P*	*P*_*adj*_	*m*_*NNC*_	*m*_*MS*_	*P*	*P*_*adj*_
Glia	−0.45	0.96	0.34	0.083	−1.17	2.5	0.0024**	0.0263*
Neurons	−1.58	3.18	0.023*	0.267	−1.6	3.2	0.0054**	0.302
Bulk	−0.93	0.66	0.24	0.059	0.04	−0.03	0.94	0.51
Blood	0.72	−0.73	0.0002***	0.0001***	0.54	−0.55	0.0068**	0.004**

Two-sided two-sample *t*-tests were performed except when comparing Horvath’s clock-derived sample means of the glial cohort. In this case, a two-sided Welch’s *t*-test has been performed to accommodate for unequal variances. m, sample mean; P*_adj_*, P-value of clinical group variable (MS/NNC) in MANCOVA with covariates sex (M/F) and PMI included (for blood cohort only sex was adjusted for as study participants were not deceased, for neuronal and glial cohort sample replicate status (TRUE/FALSE), and for the glial cohort PC1 as a proxy for different datasets of origin were added to MANCOVA), * = *P* < 0.05, ** = *P* < 0.01,*** = *P* < 0.001.

In neurons, a significant AAR difference seen with the Cortical clock did not withstand adjustment for covariates (Δ*AAR_*MS–NNC*_* = 4.8, *P*_*adj*_ = 0.302, Table 3). Similarly, Horvath’s clock did not indicate any significant difference after adjustment (Δ*AAR_*MS–NNC*_* = 4.76, *P*_*adj*_ = 0.267, Table 3). In bulk brain tissue, there was no significant AAR difference between MS patients and controls with either of the two clocks after the inclusion of covariates, although a tendency for higher AAR in MS individuals could be observed with Horvath’s clock (Δ*AAR_*MS–NNC*_* = 1.59, *P*_*adj*_ = 0.059, Figure 3F and Table 3). Stratification for MS subtypes (chronic-progressive, secondary-progressive, primary-progressive) in the bulk brain cohort yielded no significant differences either (*P*_*ANOVA, Horvath*_ = 0.24, *P_*ANOVA, Cortical*_* = 0.48). Of note, both clocks applied to blood samples showed a very significant AAR difference between healthy controls and MS patients (Table 3). Such a strong effect is expected in this cohort with a larger sample size and statistical power in comparison to our glial cohort (1-β_*Glia*_ = 0.87, 1-β_*Blood*_ = 0.96) and confirmed previous findings obtained with Horvath’s clock in the same cohort (Theodoropoulou et al., 2019).

Thus, our findings suggest that glial cells sorted from the brain of MS patients display higher age acceleration residuals compared to NNC donors.

### Sex differences in DNA methylation age

**FIGURE 4:**
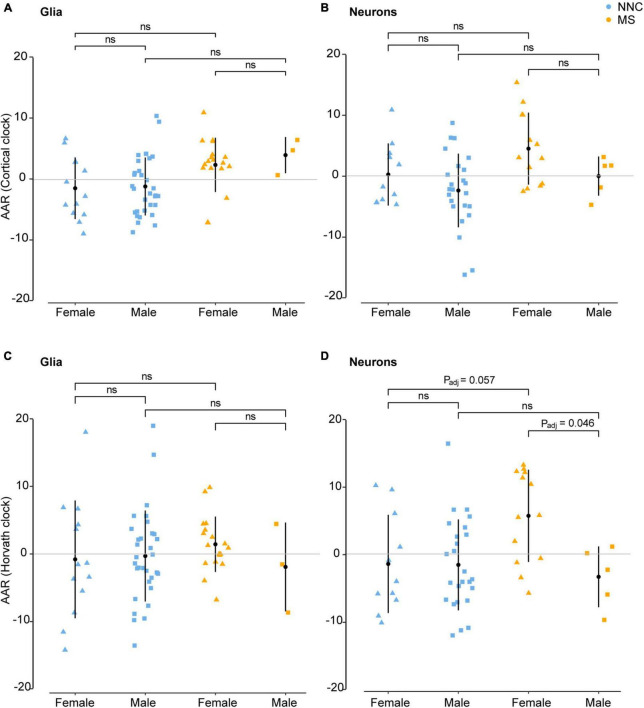
Age acceleration in glia and neurons of MS patients and controls stratified according to sex. Age acceleration residuals (AAR) in glial cells [left panels **(A,C)**] and neurons [right panels **(B,D)**] of female and male MS patients and controls were estimated using the Cortical clock [upper panels **(A,B)**] and Horvath’s clock **(C,D).** No significant sex difference was found in bulk brain tissue with neither of the two clocks. Normality was tested with the Shapiro–Wilk test. Equal variances were tested with Bartlett’s test. Sample means were compared with a Tukey HSD test, correcting P-values for multiple testing with the single-step method. Adjustment refers to PMI, replicate status, and PC1 (only for glia cohort). F, female; M, male; MS, multiple sclerosis; NNC, non-neurological controls; ns, not significant. Data were retrieved from Kozlenkov et al. (2014), Gasparoni et al. (2018), and Kular et al. (2019, 2022).

Given that MS generally affects women more often than men and considering previous reports showing that AAR varies significantly between men and women in blood samples of MS patients (Theodoropoulou et al., 2019), we examined sex-associated differences in the bulk brain, sorted neuronal, and glial nuclei. No significant sex difference was found in bulk brain tissue with either of the two clocks. In sorted cells, the Cortical clock (Figures 4A,B) did not result in significant differences between the groups after adjustment for multiple testing and covariates. While glials cell displayed no significant sex difference between clinical groups with Horvath’s clock (Figure 4C), neurons of female MS patients presented significantly higher AAR than neurons of male MS patients with Horvath’s clock (*P*_*Tukey*_ = 0.046, Figure 4D). A tendency for significantly faster biological aging was also apparent in neurons of female MS patients in comparison to neurons of female controls (*P*_*Tukey*_ = 0.057, Figure 4D).

## Discussion

In this study, we used the brain-specific Cortical clock and Horvath’s multi-tissue clock to investigate biological aging in brain-derived tissue of MS patients compared to NNC. We found a significant difference in AAR between MS patients and controls in glial cells using the Cortical clock, indicating a faster pace of biological aging in MS glial cells. Additionally, we found sex-specific differences in AAR, which were significant only in neurons using Horvath’s clock.

Glial cells are composed of highly interconnected and dynamic oligodendrocytes, astrocytes, and microglial cells that jointly orchestrate a tight control of the CNS homeostasis notably due to their pivotal roles in myelination of axons, metabolic support to neurons, and neuroinflammation. Importantly, accumulating evidence support the role of glial dysfunction and exhaustion, that is, microglial activation, failure in remyelination, and oxidative and DNA damage, among others, in the CNS inability to repair after injury typically observed at later progressive stages of MS disease (Haider et al., 2011; Fischer et al., 2013; Nijland et al., 2014). Given the vast repertoire of dysfunctions observed by glial cells in the context of progressive MS, the exact mechanisms underlying the accelerated aging observed in glial cells of MS patients remain to be clarified. Since DNAm age is known to correlate with the number of cell divisions, and thus potentially replicative senescence (Lowe et al., 2016; Horvath et al., 2018), a plausible explanation of accelerated aging in MS glia could be attributed to increased proliferation, resulting in exhausted glial cells. This exhaustion is possibly sustained by the repeated damage to oligodendrocytes and myelin (Merrill and Scolding, 1999; Miyachi et al., 2021) and the elevated demand for debris uptake by microglia (Luo et al., 2017), tissue repair by astrocytes, and remyelination by oligodendrocytes (Ponath et al., 2018). Considering the intrinsic proliferative capacity of microglia and astrocytes, higher DNAm age in glial cells could reflect increased microglial and astrocytic proliferation occurring during gliosis. This goes in line with findings from Huynh et al. showing that DNA methylation and expression changes of bulk brain tissue in MS affect genes regulating oligodendrocyte survival, proteolytic processing, and immune function in comparison to controls (Huynh et al., 2014). Furthermore, we have recently reported that glial cells of NAWM in MS patients undergo genome-wide DNA methylation changes, correlating with transcriptional differences, in genes involved in cytoskeleton organization, cell signaling, molecule transports, neuroinflammation, cell motility, and metabolic processes compared to controls (Kular et al., 2022). Altogether, this suggests that epigenetic aging of glial cells might result from enhanced cellular activity of glial cells, as reflected by DNA methylation and gene expression changes.

It is still debated whether, and under which circumstances, aging phenotypes observed in glial cells contribute to a neurotoxic or neuroprotective microenvironment, as aging does not seem to affect glial cells in the same way across brain regions. For example, during aging in mice, attenuated phagocytosis and motility of microglia could be observed in the hippocampus, whereas enhanced immune response was detected in the cerebellum (Grabert et al., 2016). Moreover, aging seems to affect oligodendrocytes proportion, particularly in the human frontal cortex, as opposed to other brain regions (Pelvig et al., 2008). However, there is a consensus that aging microglia display mitigated surveillance capacities and become more susceptible to pro-inflammatory signals, while aging astrocytes and oligodendrocytes increasingly fail to provide neurotrophic support with, for example, oligodendrocytes reducing myelination and internode length (Salas et al., 2020) and astrocytes contributing to memory defects and synaptic dysfunction (Guerra-Gomes et al., 2018). Additionally, aged rat astrocytes, cultured for 90 days, produced more reactive oxygen species than astrocytes cultured for only 10 days (Pertusa et al., 2007), a finding of relevance in the context of MS, since reactive oxygen species contribute to the demyelination of neuronal axons (Ohl et al., 2016; Papadopoulos et al., 2020). The role of aging oligodendrocyte progenitor cells (OPC) is still elusive, but recent evidence in rodents and humans suggests that OPC maturation is impaired during aging, resulting in a declining population of myelinating oligodendrocytes (Bankston et al., 2013; Papadopoulos et al., 2020; Salas et al., 2020). Moreover, findings from Yeung et al. suggested that remyelination in MS is sustained by pre-existing oligodendrocyte populations, and not by replenished oligodendrocytes originating from OPCs (Yeung et al., 2019), further adding to the notion that accelerated aging in MS could be ascribed to cellular exhaustion. Altogether, this evidence implies a lower regenerative potential of the aging CNS, which is likely responsible for the unabating decline of cognitive functions during MS disease progression.

Notably, the results of this study must be seen in the context of the limitations which are inherent in post-mortem brain studies in general and due to the scarcity of available data, particularly in MS. The small sample size of original neuronal and glial cohorts together with the limited age range and separation of younger MS donors compared to older controls prompted us to extend the age range of the participants by including additional NNC from Kozlenkov et al. (2014) and Gasparoni et al. (2018). By decreasing the lowest donor age from 35 to 18 years, we were able to reduce but not fully eliminate biases, that is, the overestimation of DNAm age in younger donors in the neuronal model using the Cortical clock (Supplementary Figure 6B). While the addition of controls from independent cohorts was not necessary to enhance the interpretability of the Cortical clock estimations in the glial cells (Supplementary Figure 6A), it enabled a gain of statistical power to identify putative differences. Indeed, it is noteworthy that a significant difference in AAR sample means between MS patients and controls could not be observed without the external control samples. Importantly, the calculation of AAR based on a model, including both MS patients and controls allowed a more conservative and unbiased approach, compared to a model using the difference between epigenetic and chronological age and/or solely control individuals, as done in other studies (Horvath and Levine, 2015; Horvath et al., 2015, 2016). Nevertheless, because the AAR values are based on linear regression, one cannot exclude the potential non-linear effect of age. Overall, our findings should be taken with necessary caution due to the modest sample size of the combined cohorts, and further cell type-restricted studies in larger case-control cohorts are warranted to replicate our findings.

While a vast number of DNAm-based clocks have emerged for the analysis of aging in peripheral blood, only limited research has so far been conducted on epigenetic measures of aging in brain tissue. With the novel Cortical clock developed by Shireby et al. (2020), DNAm age can be assessed in brain samples at an unprecedented level of accuracy measured by root-mean-square error and Pearson correlation of the chronological and predicted age. As such, Cortical clock estimations of DNAm age stand as a considerably better predictor of patients developing pathologic Alzheimer’s disease than Horvath’s clock estimations and as the sole epigenetic clock, among Horvath’s clock, Hannum’s clock, PhenoAge, and GrimAge, that significantly associated to clinical phenotypes of brain function and cognitive decline (Grodstein et al., 2021). Yet, our findings should be viewed in light of the inherent limitations of epigenetic clocks in general. These pertain, for example, to the Illumina platform used to create the clock, that is, the Horvath clock was generated using previous 450K Illumina array and 19 CpG probes are lacking on the EPIC Illumina platform. Limitations arise also from the general or specific biases in estimating epigenetic age, as observed in our data as well, which have been addressed in our study (see Materials and Methods). In our study, we confirmed that the Cortical clock provides less biased estimations of DNAm age in brain tissue than Horvath’s clock. Indeed, it does not exhibit DNAm age underestimation in donors over 60 years, as reported in previous studies using Horvath’s clock (El Khoury et al., 2019; Shireby et al., 2020), which is relevant for the study of neurodegenerative processes affecting older patients. Additionally, the Cortical clock appears less sensitive to DNAm age differences in glial cells across various brain regions in our study. Interestingly, despite using clocks trained on bulk brain tissue, differences in AAR between MS patients and NNC could be visible only in isolated neuronal and non-neuronal fractions as opposed to bulk brain tissue. It remains unclear why the AAR differences are not detected in bulk brain tissue. The limitations arising from bulk tissue analyses have already been acknowledged in a review by Bell et al. (2019), the main challenge is that the cell type-specific epigenetic mosaic becomes obscured when measuring methylation levels in heterogeneous bulk tissues. Similarly, the analysis of the NeuN^–^ fraction sorted from brain tissue, including predominantly mixed glial cell types and to a lesser extent non-glial (e.g., endothelial and peripheral immune) cells, could undeniably be biased by cellular heterogeneity. Altogether, these findings highlight the necessity to take into account cell-type specificity in both the development of epigenetic clocks, that is, training on neurons or glial cells independently, and clinical studies of the aging brain.

The difference between the results from the Cortical clock and Horvath’s clock might be attributed to biologically relevant reasons. Indeed, as only five CpG probes are shared between the Cortical clock and Horvath’s clock, different aspects of aging processes could presumably be captured by each clock, as suggested by our Gene Ontology analysis. The study of the biological underpinnings of AAR might shed light on the interpretation of surprising findings, such as the correlation between positive age acceleration and better prognosis in patients affected by gliomas with Horvath’s clock (Zheng et al., 2020) and negative age acceleration (i.e., “age deceleration”) observed in the most severely affected patients of Huntington’s disease (Horvath et al., 2016). Ultimately, it would be of particular interest to examine the association between epigenetic aging measures and clinical outcomes of MS patients, such as disease duration, disease severity, lesion burden, or cognitive abilities, which might help decipher the sex differences we observed in neurons.

## Conclusion and perspectives

In conclusion, we found that glial cells of MS patients display an increased epigenetic age acceleration compared to NNC controls with the Cortical clock. Additional studies investigating the mechanisms underlying this difference remain to be done. Because epigenetic changes are reversible by nature, future approaches, such as epigenetic-based therapies, might aid in slowing down or halting, if not reversing, age acceleration of the brain in MS.

## Data availability statement

Publicly available datasets were analyzed in this study. This data can be found here: GSE166207, GSE50798, GSE66351, GSE119532, GSE40360, and GSE106648.

## Ethics statement

The publicly available data from published studies involving human participants were reviewed and approved by ethics committees, as reported in the original publications.

## Author contributions

LK conceived and supervised the study and conducted isolation of sorted cells and DNA. DK performed data analyses and interpretation and generated the figures. AU-C performed preliminary analyses and set up an R pipeline for the data analysis. EE supervised and supported AU-C during the preliminary analysis. DG-C and NP supervised AU-C and provided biostatistical guidance. MN supervised the study, designed the analytical pipeline, and assisted DK. MJ provided overarching technical and conceptual support. DK and LK co-wrote the manuscript with contributions from all authors. All authors read and approved the manuscript.

## Footnotes

1
https://www.r-project.org

2
https://www.rstudio.com
