# Supplementary material for: Epigenetic clock indicates accelerated aging in glial cells of progressive multiple sclerosis patients
Source: Front Aging Neurosci. 2022 Aug 24;14:926468. doi: 10.3389/fnagi.2022.926468 (PMC9454196; doi:10.3389/fnagi.2022.926468)
Supplement: Supplementary file 1 [file Data_Sheet_1.pdf]

Supplementary Material: Kular, Klose et al. Epigenetic clock indicates accelerated ageing in glial cells of progressive Multiple Sclerosis patients

Table S1 | Number of remaining CpG probes after quality control used for *agep()* DNAm age calculation.

	Bulk brain		Glia		Neurons		Blood	
Clock	*Cortical*	*Horvath*	*Cortical*	*Horvath*	*Cortical*	*Horvath*	*Cortical*	*Horvath*
N Probes after QC	345	353	347	333	347	353	347	353
Percentage covered	99.1%	100%	99.7%	94%	99.7%	100%	99.7%	100%

N = number, QC = quality control.

Table S2 | Shared and clock-specific pathways of CpG-annotated genes.

Shared	Horvath clock	Cortical clock
Pathway	*Pathway*	*Pathway*
fatty acid degradation	transport of small molecules	cellular aldehyde metabolic process
major receptors targeted by epinephrine + norepinephrine	regulation of lipid catabolic process	valine, leucine and isoleucine degradation
monocarboxylic acid metabolic process	plasma lipoprotein assembly	renin secretion
regulation of lipid metabolic process	regulation of lipase activity	regulation of muscle system process
regulation of tube diameter	beta-alanine metabolism	lipoxygenase pathway
amp metabolic process	response to inorganic substance	carbon metabolism
response to hormone	negative regulation of catalytic activity	inflammatory response
multicellular organismal homeostasis	regulation of hormone levels	response to extracellular stimulus
response to alcohol		
response to xenobiotic stimulus		
thiamine metabolism		
regulation of secretion		

The 20 most significant pathways are shown, sorted by Benjamini-Hochberg corrected -logP-value (-logP range: 30.2 – 9.4). Analysis has been performed with Metascape Gene Ontology. For the Cortical clock only 82% of CpGs yielded an annotated gene, for Horvath's clock 99% were annotated (Reference genome: hg19). To map CpG sites to gene names, the *getAnnot()* function from the *methylGSA* package was used.

Supplementary Figure 1

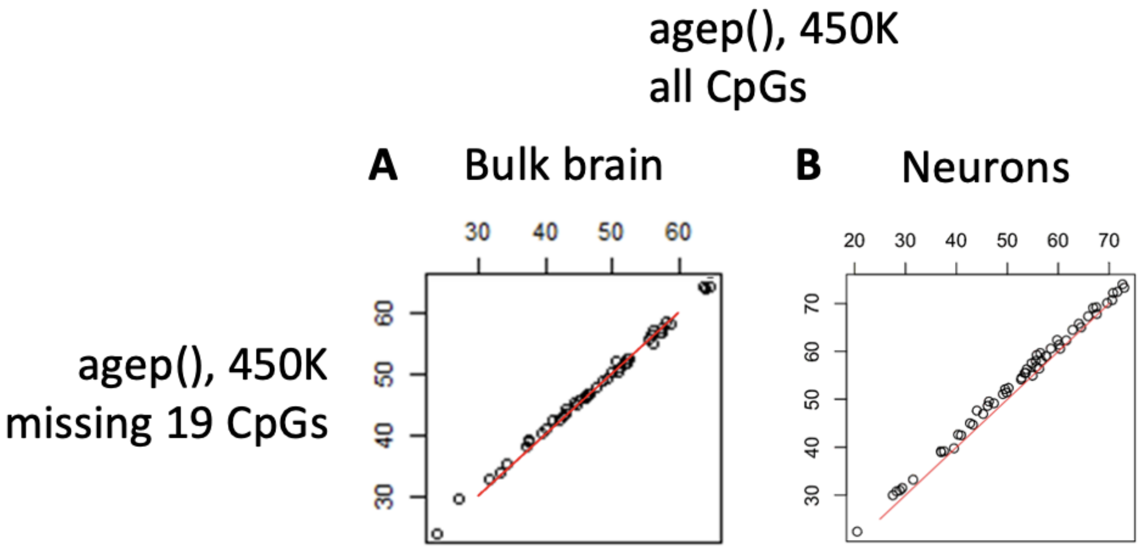

Figure S1 | The 19 missing EPIC CpG probes have no strong effect on DNAm age estimation with Horvath's clock on the 450K array. For this analysis, 450K data from bulk brain data (A) and neuronal data (B) were used. A trend towards systematic overestimation of epigenetic age could be observed when 19 CpG probes were missing in the sorted neuronal cells. Red line denotes perfect correlation between prediction with and without 19 missing EPIC probes.

Supplementary Figure 2

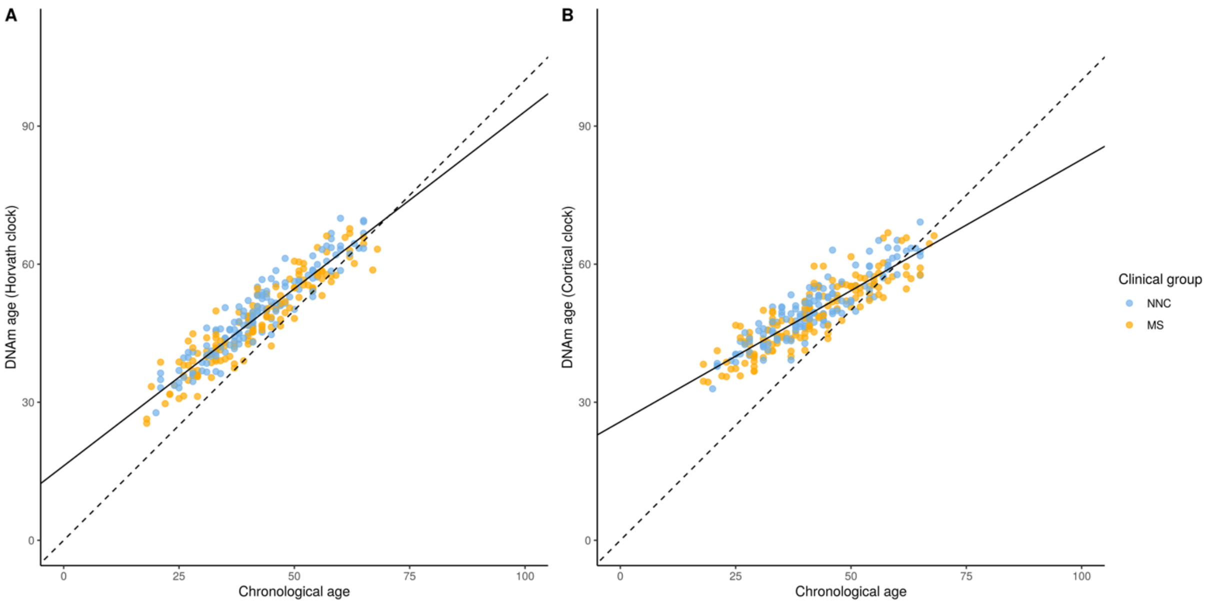

Figure S2 | Regression of estimated DNAm age on chronological age in whole blood cohort.
(A) Underestimation of DNAm age by Horvath's clock for older age groups is not observed in whole blood. However, the estimation is biased for younger age groups. **(B)** The bias for younger age groups is even stronger when using the Cortical clock for the whole blood data.

Supplementary Figure 3

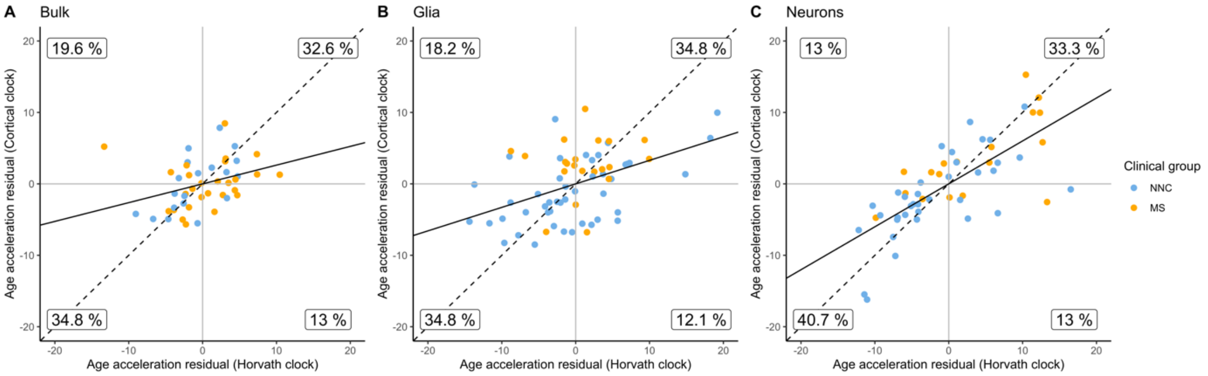

Figure S3 | A fraction of samples receives discordant estimations by the two clocks.
Approximately one quarter of samples are placed in the second or fourth quadrant of the coordinate system, meaning that one clock predicts positive AAR, whereas the other predicts negative AAR. In the neuronal cohort, Horvath's clock and Cortical clock have the most accordant estimations. Legend: Black line represents linear regression line, dashed line represents the x=y bisector, plot labels represent percentage of data points in the respective quadrant. **(A)** Bulk brain cohort: Pearson's $r = 0.33$, $P = 0.022$ **(B)** Glia cohort: Pearson's $r = 0.46$, $P = 0.0001$ **(C)** Neuronal cohort: Pearson's $r = 0.72$, $P = 6.87\times10^{-10}$. Data was retrieved from (22-26).

Supplementary Figure 4

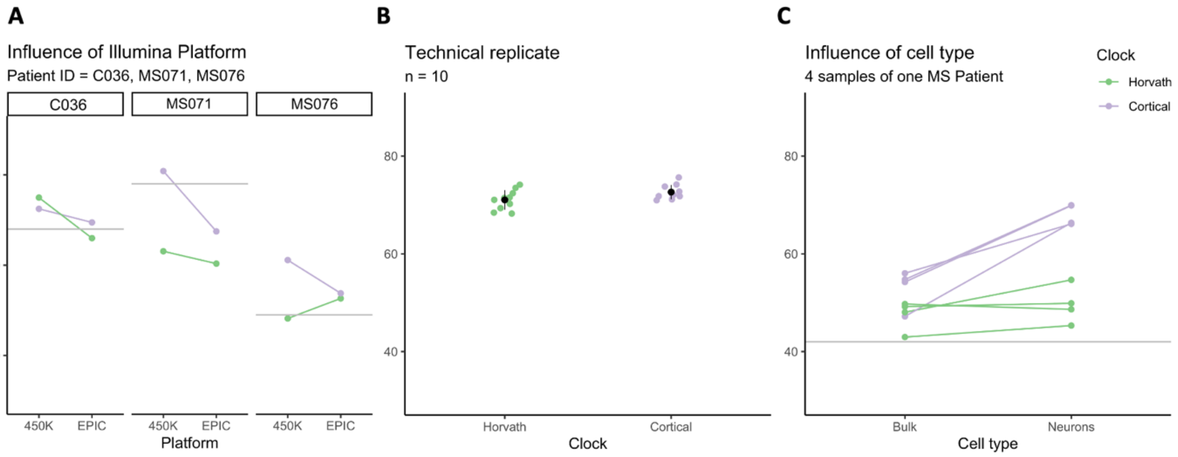

Figure S4 | Influence of confounding factors on DNAm age estimation.

(A) Influence of Illumina 450K or Illumina 850K (EPIC) on estimation of DNAm age. Shown are estimations for the same neuronal samples, both on 450K and on EPIC. **(B)** Technical replicate placed on different EPIC slides. The array position was fixed. **(C)** Comparison of estimations in bulk brain tissue and sorted neurons from the same sample with both clocks. Legend: Gray line shows chronological age of the analysed donor. Data was retrieved from Kular *et al* (2019).

Supplementary Figure 5

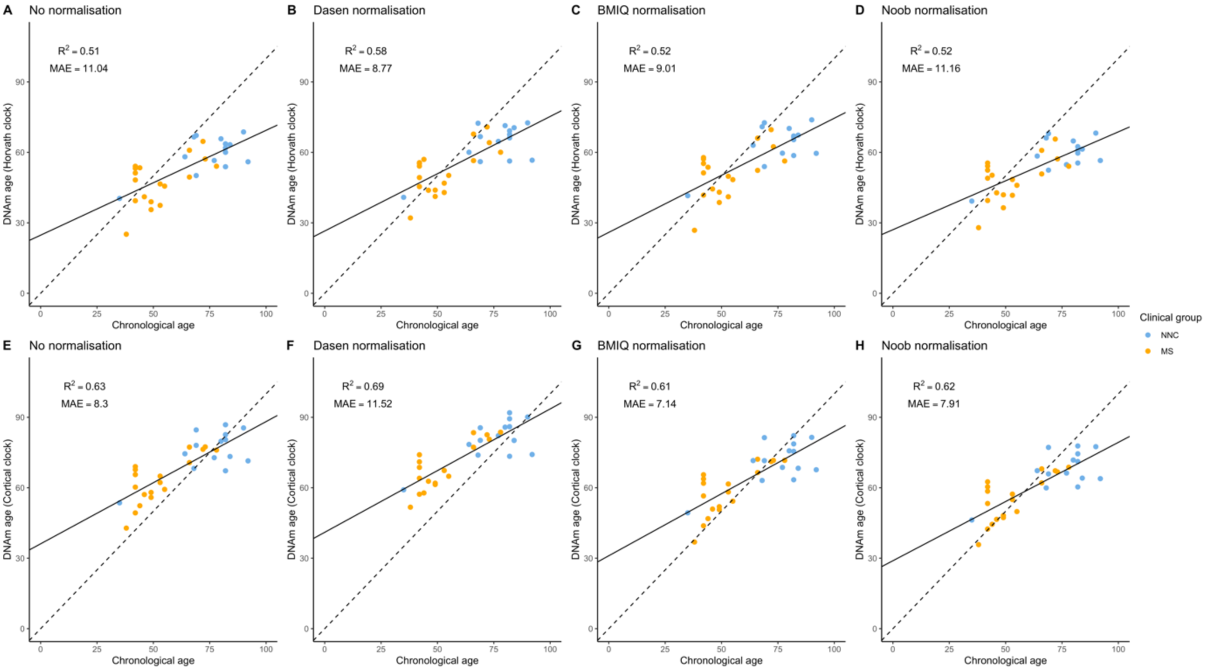

Figure S5 | Dasen normalization yields best linear model for both Horvath's clock and Cortical clock derived DNAm ages.

(A, E) No normalization was applied to the beta values. **(B, F)** Dasen normalization was applied to the beta values. **(C, G)** BMIQ normalization was applied to the beta values. **(D, H)** Noob normalization was applied to the beta values. Raw data was retrieved only from Kular *et al* (2019) as Noob normalization required an idat-file derived data format, which were not provided by all other studies.

Supplementary Figure 6

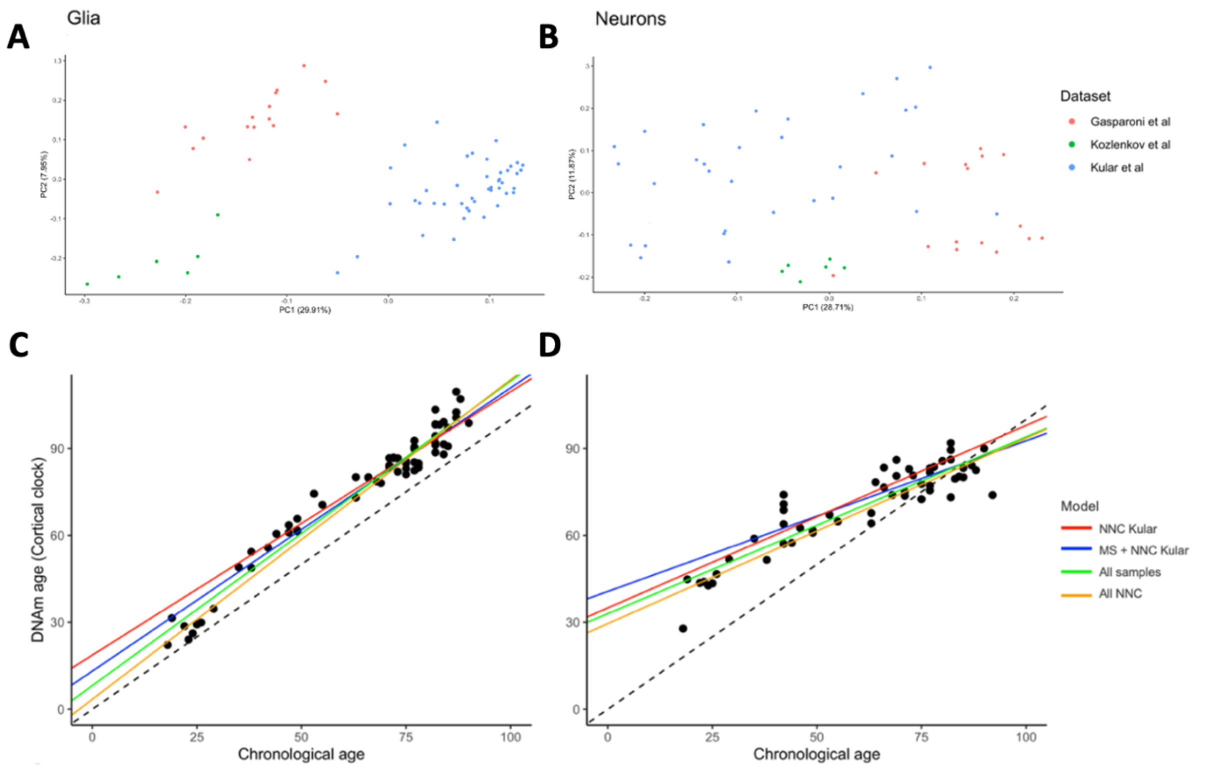

Figure S6 | Influence of the datasets on the linear models of the glial and neuronal cohorts. Principal component analysis of 441 330 autosomal CpGs for the glial cohort **(A)** and 482 946 autosomal CpGs for neuronal dataset **(B)** from both MS patients and NNC. Regression lines of datasets used for the glial **(C)** and neuronal **(D)** cohorts. Red line represents only controls from Kular *et al*, blue line represents MS patients and controls from Kular *et al*, green line represents all MS patients and controls, orange line represents all controls. MS = multiple sclerosis, NNC = non-neurological controls. Data was taken from (Kular *et al* 2019, 2022; Kozlenkov *et al* 2014, Gasparoni *et al* 2018 and Huynh *et al* 2014).
